# The Need for A Training Software among Iranian Infertile Couples: A Qualitative Study

**DOI:** 10.22074/ijfs.2019.5727

**Published:** 2019-04-27

**Authors:** Zahra Haji Naghib Ali Hesari, Razieh Lotfi, Behrooz Pouragha, Bita Badehnoosh, Mansoureh Yazdkhasti

**Affiliations:** 1 Department of Midwifery, Student Research Committee, Student of Midwifery Counseling, Medical School, Alborz University of Medical Sciences, Karaj, Iran; 2 Department of Midwifery, School of Medicine, Alborz University of Medical Sciences, Karaj, Iran; 3 Department of Public Health, School of Health, Alborz University of Medical Sciences, Karaj, Iran; 4 Department of Obstetrics and Gynecology, Dietary Supplements and Probiotic Research Center, Alborz University of Medical Sciences, Karaj, Iran; 5 Department of Midwifery, Faculty of Midwifery, Social Determinations of Health Research Center, Alborz University of Medical Sciences, Karaj, Iran

**Keywords:** Infertility, Knowledge, Qualitative Research, Training Programs

## Abstract

**Background:**

Training needs are multidimensional requirements affected by social and cultural background, level of knowledge and personal and health conditions. This study was conducted to explain the needs for a training software among Iranian infertile couples.

**Materials and Methods:**

In this qualitative study, we used content analysis to examine the need among ten infertile participants (four men and six women) and six health care professionals (including two gynecologists, two reproduc- tive health specialists and two midwives). The present research was carried out from January 2017 to July 2018 at Rouyesh and Ibn Sina infertility treatment centers in Karaj, Iran. The participants were selected through purposive sampling with maximum variation. Four focus group discussions with the health care professionals and twelve semi- structured, in-depth interviews with the infertile participants were held for data collection. Data were analyzed using conventional content analysis in MAXQDA-10.

**Results:**

Data analysis led to the extraction of a central theme of “a multidimensional training application” and its four main categories, including "pre-treatment training", "diagnostic training", "mid- and post-treatment training" and “continuous psychological training". These main categories also had 20 subcategories.

**Conclusion:**

Based on the results of this study, infertile women and men have multidimensional training needs before and after treatment and during the process of diagnosis; psychological aspects should also be considered.The inter- viewed health care professionals helped to explain these training needs. A training software thus needs to be designed based on the real needs of the infertile community.

## Introduction

Adult training programs are developed based on the priorities of the learners’ needs (1-3). Meeting the population’s diverse and changing health needs is the most important mission of health education organizations. If the training programs developed by these organizations are based on the needs of different target groups of different social, economic, cultural and psychological backgrounds within the community, they can effectively improve the community’s health (4). The infertile population is a target group that requires an effective training program for their condition. Having children is one of the most basic needs in married people’s lives and forms the basis of all human life (5-7). Infertility is “a disease of the reproductive system defined by the failure to achieve a clinical pregnancy after 12 months or more of regular unprotected sexual intercourse”. The condition is divided into primary and secondary types. There is no pregnancy in primary infertility, while one or more previous pregnancies existed in those with secondary infertility. Couples with secondary infertility were able to conceive at least once, but are now unable to conceive again (4).

The current rate of primary infertility is estimated 2.8% by the National Health Survey (NHS) and 3.4% by the National Infertility Survey (NIS). NIS conducted a survey of 12,000 women aged 19 to 49 years in all 28 provinces of Iran in 2004-2005. A study in Tehran and also the NIS report estimated the prevalence of lifetime primary infertility to be 21.9 and 24.9%, respectively. The minimum prevalence of lifetime primary infertility was found to be 15.8% for the marriage age of 19-27 years in the study conducted in Tehran. Some studies in Iran reported that an average of 21-22% of women experience primary infertility during their marital life (3).

As a significant multidimensional issue, infertility can affect couples’ physical, emotional, sexual and social health (5). One of the issues which infertile couples would face is lack of awareness about multiple dimensions of dealing with infertility. Some of these dimensions include diagnosis and treatment. The lack of awareness and training causes more confusion and psychological stress for infertile couples and leads to more complex medical treatments, higher financial costs and prolongation of treatment process (2, 3).

The first step in educational interventions is understanding the training needs of the target population. The final process of training pertains to the correct implementation of the needs assessment before designing the training program. Needs assessment is the process of collecting and purposefully analyzing data for identifying the needs of individuals, groups, organizations and communities; before presenting or prioritizing the obtained information, it is essential to get the patients’ actual needs (i.e. the gap between the status quo and the desired situation) as assessed by health care professionals (8).

Using qualitative methods is a way to understand the needs of infertile couples. Quantitative approaches fail to process complex multidimensional concepts. Quantitative research often involves limited variables and does not reveal the depth of the existing reality. Therefore, conducting qualitative research would result in a better understanding of the needs of infertile women and men (8-10). Content analysis is a qualitative research technique used to make valid replicable inferences by interpreting and encoding text. Qualitative content analysis is suitable for obtaining valid results as text data for generating knowledge, new ideas, facts and guidelines for proper performance (11). This method is used to interpret and classify textual data by considering the cultural and environmental aspects affecting the studied phenomenon (12, 13). In Iran, Karaj is considered the “tiny Iran”, since it is composed of several demographic groups of different cultures. This city offers limited resources to infertile couples. It is therefore beneficial to develop an application based on patients’ educational/ training needs.

Needs comprise a multidimensional phenomenon (5) affected by the socio-cultural context in place. Very few studies were done on the training needs of the infertile population in the country, and none them examined the training needs of infertile couples with respect to development of an application. Although some qualitative studies on infertility explained the needs of infertile couples, the present study, for the first time, explored their needs in terms of having access to a software application. The main question of the present study is ‘What types of training do you expect from an application to provide? In other words, what should such an application include to be able to increase your knowledge and awareness about your areas of need?’ This study was thus conducted to discover the training needs of infertile couples to be considered when designing a software application based on the explored real needs of infertile couples and health care professionals.

## Materials and Methods

### Participants and data collection

Four focus group discussions (done by six health care professionals) and 12 semi-structured interviews (held with ten infertile participants) were completed in this study on a total of 16 participants. The inclusion criteria included: i. Being an infertile man or woman or an infertility healthcare provider, ii. Having Iranian nationality, iii. Having at least reading and writing nationality, iv. No mental disorders based on the self-report, and v. The healthcare providers and clinicians with at least two years of work experience were taken as the group of health care professionals.

This qualitative study used a content analysis approach to examine ten infertile participants (four men and six women) and six health care professionals (two gynecologists, two reproductive health specialists and two midwives), from January 2017 to July 2018 at Rouyesh and Ibn Sina infertility treatment centers, Karaj, Iran.

First, based on the health records available at these two infertility treatment centers, 42 infertile couples were contacted by text message and invited to attend a briefing session at a specified time and place. A total of 25 people showed up. Twenty of the attendees volunteered to take part in the study. Then, based on the inclusion criteria, ten infertile couples entered the study. Six health care professionals including two gynecologists, two reproductive health specialists and two midwives, with at least two years of work experience in infertility treatment, were also recruited. The participants were selected through purposive sampling with maximum variation. Four focus group discussions were held with the health care professionals and 12 in-depth semi-structured interviews were held with the infertile participants for data collection. It should be noted that two out of ten infertile couples were interviewed twice due to their higher knowledge and experience about infertility; therefore, a total of 12 interviews were ultimately held with the couples.

This study examined the training need of Iranian infertile couples and health care professionals with respect to development of a training software or application. The main questions of the study were "What are the training needs of infertile women and men regarding their infertility?" and "What are the training needs of infertile women and men that should be considered when developing an application?" The interviews were tape-recorded with participants’ permission. The interviewer took field notes immediately after each interview. All interviews were held by corresponding author (M.Y) after approval of all research team members. The interviews lasted from 40 to 60 minutes, with an average duration of 50 minutes. The average time for the four focus group discussions was 75 minutes.

The infertile participants were selected through purposeful sampling. One type of purposeful sampling is variation sampling method. In this study, variation sampling was considered in terms of age, gender, education, occupation, socioeconomic status, duration of marriage, duration of infertility and cause of infertility (Table 1). The health care professionals were selected through the same method, with variation in terms of gender, education and work experience (Table 2).

**Table 1:** Demographic characteristics of the infertile couples


Variable		n	Variable	n

Age (Y)			Socioeconomic status	
	25-30	5	Good	4
	31-35	2	Moderate	3
	36-40	3	Poor	3
Sex			Duration of marriage (Y)	
	Female	6	1-5	6
	Male	4	6-10	2
			11-15	2
Education level			Time of infertility (Y)	
	Diploma	4	1-5	7
	Bachelor	3	6-10	2
	Masters	3	11-15	1
Job			Cause of infertility	
	House keeper	5	Female factor	5
	Employee	3	Male factor	2
	Informal Job	2	Unexplained	3


**Table 2:** Characteristics of the health care professionals participated in the present study


Variable		n

Sex		
	Female	5
	Male	1
Education level		
	Specialty	2
	PhD	2
	Masters	1
	Bachelor	1
Work experience (Y)		
	1-10	3
	11-20	3


The individual interviews and focus group discussions were held at the time and place of participants’ choosing. Table 3 lists some of the interview’s questions. Probing questions were also asked, such as “Can you elaborate on that/provide an example?”

**Table 3:** A sample of interview questions



1. What are your training needs with regard to your infertility? (infertile couples)
2. Based on your experience, what features should a well-designed educational computer application have to have in order to meet the needs of infertile people? (infertile participants)
3. How do you suit your training needs regarding infertility? (infertile couples)
4. What are your training needs regarding infertility? (infertile couples)
5. What questions do you tend to ask infertility health care professionals to meet your training needs? (infertile couples)
6. What questions are more frequently asked by your patients about infertility diagnostic-therapeutic steps? (health care professionals)
7. Based on your experience, what kinds of training needs do couples have regarding infertility? (health care professionals)


### Data analysis

Data were analyzed using the method of data analysis provided by Graneheim and Lundman (14); the following steps were taken to analyze the collected data: i. Transcribing the interviews verbatim and reading through several times to get a general sense of the material, ii. Dividing the text into meaning units, which are key phrases in the text, iii. Abstracting the condensed meaning units and outlining using codes, iv. Grouping codes into sub-categories and categories based on comparisons made based on similarities and differences, v. Re-organizing and merging into sub-themes and overarching themes as the expression of the latent content of the text participant.

MAXQDA-10.0 was used to facilitate data coding, categorizing and retrieval. This tool allows to analyze the combination of activated codes in different ways, taking into account the different groups and positions of coded segments. It helps to retrieve and arrange codes in a category.

### Trustworthiness of the data

To ensure the credibility of the data, feedback was obtained from the participants (member check) and the number of interviews with some of the participants was increased. To increase the transferability of the findings to other settings and groups, the participants were selected with maximum variation and various experiences in terms of the study subject. Also, the confirmability and dependability of the findings were established through peer check, peer debriefing, review of the transcripts by some of the participants, researchers’ immersion in the study subject and prolonged engagement with the data.

### Ethical considerations

This project was approved by the Ethics Committee of Alborz University of Medical Sciences (IR-ABZUMS.REC.1397.032). All the participants were briefed on the objectives and methods of the study and they signed informed consents. Before the interviews, the participants were ensured that they have the right to refuse to participate in the interviews at any time without any negative consequences for the services they receive. The authors have fully observed the ethics of research, including submitting informed consent and avoiding plagiarism, misconduct, data fabrication and/or falsification, double publication and/or submission, redundancy, etc.

## Results

**Fig 1:**
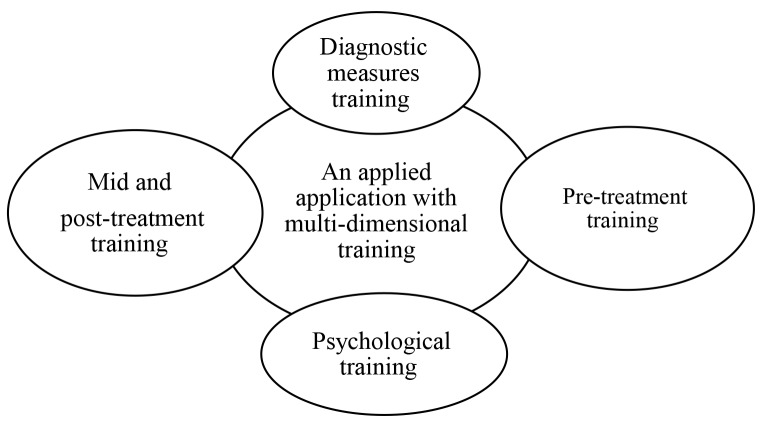
The relationship between the theme and the main categories.

Tables 1 and 2 present participants’ characteristics. After analyzing the data, the findings were explored in one theme, entitled "A multidimensional training application". The theme includes four main categories (Table 4) namely, "pre-treatment training" (with ten subcategories), "diagnostic training" (two subcategories) "mid- and post-treatment training" (five subcategories) and "continuous psychological training" (three subcategories), and 130 codes (from originally 658 primary codes). Figure 1 presents the relationship between the theme and the main categories.

**Table 4:** The theme, main categories and subcategories


Theme	Main category	Subcategory

A multidimensional training application	Pre-treatment training	The fertility process
		The causes of infertility
		Assisted fertility techniques
		The process of transfer in assisted fertility techniques
		Pre-treatment nutrition
		The necessity of scientifically-filtered training
		Understanding infertility by visiting the web
		Access to valid infertility centers
		Pre-treatment medications
		Ethics
	Diagnostic training	The step-by-step of diagnostic measures
		Learning effective measures for improving the quality of spermogram
	Mid- and post-treatment training	Mid- and post-treatment medications
		Mid- and post-treatment self-care
		Mid- and post-treatment complications
		Mid- and post-treatment nutrition
		Mid- and post-treatment feedback
	Continuous psychological training	Stress management strategies in diagnostic and treatment procedures
		Mental relaxation
		Strategies for improving self-confidence or self-esteem


### Pre-treatment training

The first step in maintaining future fertility is awareness about the risk factors of infertility. The majority of the participants of this study prioritized the design of a training program that covers infertility issues and trains couples about their fertility health, although they receive some sort of training on these subjects in different contexts and at various times, they were seeking unlimited access to more comprehensive written information. Further access to this information would make them examine their fertility health and infertility issues sooner, and help them begin treatments on time. Participant 3, who was infertile, made the following comment:

“When we are informed about the natural process of fertility and the things needed for its success, we start looking for treatment sooner”.

Considering the causes of infertility, the participants believed that couples need to know the exact causes of their infertility to better deal with their particular case of infertility. Participant 4, also infertile, commented:

"I think that the causes of infertility should be fully explained in a simple and accurate manner, so they can be easily understood; for example, your infertility is caused by these four reasons and you are lacking in these four areas. I get a lot of my information on the web, but I like to get information from a valid source as well".

A training tailored to the cultural context of the society in question and offered in a simple and easily-comprehensible language helps to improve couples’ process of infertility treatment. According to the participants, awareness about the available methods of treatment can help to choose the correct infertility treatment and reduce the errors. Participant 6 stated:

“Before beginning any type of treatment, we gather a lot of accurate information to be able to choose the correct route faster. A software application is way better, since it is always available on your cellphone, offers you precise information and reduces the probability of error".

The other participants discussed their need for knowledge about the reasons for their treatment failure. Participant 2, also infertile, said:

"In case of a potential treatment failure, for example, there should be a brief explanation of the issue to satiate your curiosity and prepare you for upcoming events."

The need for proper nutrition training was another significant component of infertility pre-treatment that was considered a necessary part of the training software by the participants. Participant 4, also infertile, said:

“It is better that the software include some sort of diet that help to improve your treatment outcome and follicle growth during the treatment period, since our information is limited".

One of the health care professionals (a gynecologist) said:

"A poor long-term diet disrupts the body’s physiology. For the patients who do not have access to a nutritionist, you can suggest a brief simple diet to follow. For example, the number of calories needed in the main meals can be stated".

According to the participants, advanced technologies are a step toward training services with a higher quality, greater validity and less damage to the environment. Participant 8, also infertile, said:

"I always wonder whether these websites are valid or not. This way, the need for paper is reduced, and it is good in terms of environmental protection. I think the software application can provide better training with better quality".

The participants said that the training application is a way to enhance their awareness and contributes to their readiness to move along with their infertility treatment procedures. Participant 3, who was infertile, said:

"Awareness empowers couples in terms of knowledge and leads to a better understanding of the issue. A well-informed and aware person is always successful".

The participants also needed pre-treatment training on ovum donation concerning family and religious issues. For example, some couples were worried about the similarity of their future child’s appearance to themselves and the religious issues concerning the donor family. In this respect, participant 3 (infertile) stated:

"For example, when they say that they give you another woman’s ovum, I want to know more about it. How exactly does it work? Is the donor a good person? I do not know her. Can I get involved in this procedure? What is the Islam’s point of view on this subject?"

### Diagnostic training

Based on the experiences of the participants, a step-by-step training process can help with diagnoses. Participant 6 (infertile) said about diagnostic training:

"Why does each diagnostic process have to be carried out? For example, what is a Doppler sonography? How does it work? How does it help to make a diagnosis? I assume it would be better if these were explained one by one and step by step."

Explaining the prerequisite conditions of diagnostic measures was also considered necessary for reducing the frequency of test repeats. One gynecologist stated:

“They mostly ask if it is OK to provide the samples at home or not. Well, if the samples are left in the open air for more than 20 minutes or half an hour, or if they are not kept under appropriate storage conditions, the test results would be affected. If the patients understand the significance of speed in these processes, they can cooperate more easily".

### Mid- and post-treatment training

Many infertile couples experienced the need for mid and post-treatment training in many aspects. One of these needs is the timing of the prescribed medications. Participant 5 (infertile) said:

"I even set the alarm, but I am still worried about not waking up. Now, it would be great if something like a medication alarm could remind us of the time I should take my medicines".

 Training on self-care behaviors is crucial for couples who have various diseases (15, 16). Mid- and post-treatment self-care was another need discussed by the participants in this study. Participant 4 (infertile) said:

 “Couples are often stressed, but if they knew what to do and how to take care of themselves, it would be better. It would be quite useful if your training program, in a simple language, explains what self-care measures are necessary to take after infertility treatment".

### Continuous psychological training

The experience of infertility imposes considerable psychological pressure on infertile couples in the socio-cultural context of the society. Based on participants’ experiences, the need for psychological training is great in infertile couples. The need for training on how to achieve mental relaxation, increase self-esteem and cope with stress was a part of participants’ psychological needs. Participant 5 (infertile) said:

"Well, couples tend to emotionally collapse in such situations. It is more helpful if this app also incorporates training on how to calm oneself down. This way, better results can be expected for infertility treatment".

One of the infertile male participants said:

"It would be really good if there was training about how to repel or reduce negative pressures somewhere in the app".

Another infertile male participant said:

“My wife has no fertility problems; sometimes, I blame myself, and wish we had not got married. Negative thoughts put a lot of pressure on me and it would be helpful if there were strategies to get me more relaxed. For example, I try to calm myself down with praying and saying prayers. I need to learn some other strategies too".

In this regard, a gynecologist at the infertility center said:

"Many cannot attend the classes in person. Now, if these items are included in the app, they can have a positive effect on all patients and keep the negative thoughts away".

Supporting the infertile spouse in the process of dealing with infertility was another need discussed by the participants. Participant 7 stated:

 "I did not receive emotional and mental support from my husband’s family; however, my husband himself is always besides me, gives me positive energy and supports me when I get tired of taking my medications. His behavior is so inspiring for me to keep up with this whole thing and not get disappointed".

## Discussion

Multidimensional training is a fundamental need for promoting health and improving the quality of life. The training needs of infertile people are derived from their cultural, social and religious background (9, 10).

This qualitative study explains the training needs of Iranian infertile women and men and health care professionals working in the field, for development of a training software program. Based on previous studies, infertility training needs require more efficient and up-to-date methods. In today’s world, e-learning has addressed many of these training challenges, such as the challenges of formal training, which requires a physical location, training facilities and instructors (17). During the period of infertility treatment, there is a considerable lack of awareness about the pre-, mid- and post-treatment measures as well as the medical and therapeutic measures that should be taken, which doubles the difficulty of this period. Easy access to information and training materials through the internet facilitates reaching a larger number of audiences in different environments. Providing information about infertility through software programs, enables couples to have easier and more extensive access to the information (7). Poor knowledge and inadequate awareness about fertility health reveals the need for further training. The participants, in the present study, noted the need for more training about fertility health and the reasons for infertility as parts of the reason for delaying their infertility treatment. In a study conducted by Kudesia et al. (18), the need to train women and men was emphasized due to the inadequate knowledge about fertility health. During their reproductive years, 55.9% of men and women lack adequate knowledge about fertility. Such knowledge gap has a direct relationship with the delay of infertility treatment and the delay in its start. Awareness about the problem was discussed by 40% of the participants as the first step in seeking infertility treatment. The participants emphasized the need for being trained about fertility health and the reasons for infertility through training programs. In another study, Swift and Liu (19) reported a level of knowledge of 49.9% about fertility health in infertile women in Canada. This research demonstrated women’s need for more training on fertility health issues.

The participants of the present study needed to be trained on a variety of diagnostic measures and infertility treatments. They considered using the software application as a reliable and valid method for getting answers to their questions. A total of 92.2% of the subjects in the study done by Hampton and Mazza (20) stated a need for more infertility training programs to learn the causes of their infertility and various diagnostic and therapeutic measures they had to undertake in a simple and comprehensible written form for better dealing with the issue of infertility. The participants in the present study were also concerned about collecting information and becoming aware about fertility or infertility issues as they sometimes received contradictory or invalid information through invalid resources. They needed scientific information in the form of a software application. Conceição et al. (21) conducted a study and found that infertile men and women suffer from poor knowledge on their health issues and have contradictory infertility information. The study concluded that these groups needs more valid information on fertility and infertility health issues. Valid e-learning training was therefore used due to its ease of access and scientifically-filtered information. After this e-learning intervention, the subjects were reported to have higher knowledge about fertility health and infertility, and a statistically significant increase was reported in their knowledge and awareness.

Many questions were posed by the participants about diagnostic and treatment measures, which increased the personnel’s workload in infertility treatment centers, and the software application was noted by them as a better way to answer such questions. In the study done by Hampton et al. (22) in Australia, time limits were reported by the subjects as the greatest barrier to training the staff and physicians in infertility centers. Inappropriate training materials were also noted to cause a significant gap in providing primary care to infertile women. The participants considered the need for training on fertility and infertility as one of the main steps in infertility pre-treatment that reduces their mental stress and anxiety (22-24). In this regard, Dawdy et al. (23) reported multimedia information as a method for minimizing the waiting time for the patients and improving their awareness. They also reported that using appropriate training tools can improve treatment outcomes and reduce the costs of hospitalization. In other words, in their study, the readiness of the patients, obtained through multimedia training, reduced stress and anxiety.

In the present research, the participants stated that a training software program is an appropriate way for increasing awareness and empowerment. In the study entitled "The theoretical framework of women's health in Iran: The Farmehr model", security in the family, the family dimension and composition, accessing and utilizing material and spiritual resources, and social capital, social support, social networks, public trust, social norms and social actions contributed to women’s health and formed a sense of power/empowerment or powerlessness in them (25). In another study conducted in Iran, Mohamadirizi et al. (26) trained health care providers and reported that pamphlets or training materials alone cannot always meet the different levels of learners’ expectations. In their study, using e-learning led to an increase in satisfaction and improved the level of learners’ knowledge.

The other needs raised by the participants included self-care and infertility treatment training, which should be included in the training software application in a simple comprehensible language. The module contained authentic and scientific information with a simple and comprehensible language to help patients pursue self-care (27). The results showed that e-learning increases the level of knowledge and improves self-care and self-efficacy in patients (28).

Infertility was described by the participants as a reason for their mental and psychological pressures that exposed them to contempt in the society and made them ask for psychological training and its inclusion in the training software application. In the Iranian culture, men’s conception of a woman is a significant construct in the image she has of herself, i.e. her self-concept (29). The childbearing and motherhood role is the centerpiece of the role of women in traditional societies. With this social perspective dominating societies, the threat to femininity is more manifested in infertile women and causes more damage to women’s identities and leads to their humiliation and lower self-esteem (9, 29, 30). The participants who experienced more support and attention from their spouse were better able to calm down and face their infertility. In their mixed study, van Empel et al. (31) reported the lack of emotional support as a considerable weakness in infertility treatment. They considered the involvement of the spouse in the other partner’s care a significant source of positive energy that helps to improve the treatment outcomes by reducing psychological stress.

In recent years, medical sciences have emphasized the relationship between infertility and psychological factors (29). Rahmani Fard et al. (6) argued that by eliminating or reducing the feelings caused by infertility, mindfulness exercises can make a significant contribution to the quality of life of this group of women. They concluded that training infertile women with mindfulness techniques can help them to improve the physical and mental health.

Resorting to worship, saying prayers and praying were measures that had helped the participants to achieve mental relaxation. Spiritual measures pushed away any negative thoughts and they emphasized the importance of including spiritual stress management techniques in the training software application to be able to go through the complex and sometimes annoying stages of coping with infertility. The interconnection between the body and the mind is irrefutable, such that the body-mind relationship can associate any natural or psychological change in life to psychological symptoms and cause adverse health effects (32). Praying gives the individual spiritual agency, and alongside material factors, plays an effective role in controlling, managing and adapting to the complex issues of life and the treatment of diseases. Prayer is a type of meditation in which the person joins the Divine through connectivity to the extreme energy. The belief in a superior strength pushes away person’s sense of loneliness and helplessness. Praying also helps the person to find a partner for sadness, talk about their uncomfortable thoughts and think deeper about the issue. In other words, prayer acts as a source of inspiration for the person to find power and ability to continue his efforts. In a qualitative study done by Soleimani et al. (32), spiritual care was proposed as a concept experienced by patients with Parkinson’s disease. Religious beliefs and practices such as praying and saying prayers were among the coping strategies that most Parkinson’s patients used to achieve psychological peace of mind and stability and resistance against illness. Spiritual health therefore plays a significant role in mental health and can facilitate adaptation to health crises and health-threatening conditions. There is also a relationship between spiritual health and the symptoms of infertility-induced depression and stress (33). The results of a study done by Mehrabi et al. (33) showed that there is a direct and significant relationship between the score of spiritual health and the quality of life. To justify this finding, they stated that couples with chronic illness are likely to suffer from stressful social and psychosocial stresses, such as existential conflicts related to meaning and purpose, and suffering from illness often challenges the meaning and purpose of life too. Adopting religious practices can thus help to manage thoughts and feelings and improve the quality of life.

A limitation of this study was its small sample size. Also, similar to all qualitative studies, its results are not intended to be generalized, although maximum variation sampling was used.

## Conclusion

Based on the results of this study, infertile women and men have multidimensional training needs before and after treatment, and during the process of diagnosis; also, they need psychological supports. These concepts were extracted based on the real needs of these couples. The health care professionals also elaborated on these needs. Designing a training software based on the real needs of the infertile community thus seems essential and can partly cover the shortage of training staff employed in infertility centers. Based on the findings of this study and the themes extracted from them, the researchers found that the design of this application must incorporate main themes such as fertility (proper explanation of the inner and outer genital system), diagnostic measures (proper explanation of tests such as blood tests, proper sampling time and requirements of the spermogram), infertility treatment [proper explanation of intrauterine insemination (IUI), *in vitro* fertilization (IVF) intracytoplasmic sperm injection (ICSI) and their complications] and psychological and mental health issues experienced by couples using appropriate skills, such spiritual care and mind control. The researchers recommend more qualitative studies to be conducted on the training needs of particular infertile groups, such as infertile patients with multiple sclerosis, spinal cord injury or recurrent abortion, to design better educational content on infertility.

## References

[1] Kabeer N (1999). Resources, agency, achievements: reflections on the measurement of women's empowerment. Development and Change.

[2] Hossein Rashidi B, Malek Afzali H, Haghollahi F, Abedini M, Eslami M (2017). The utilization of infertility services by infertile couples in Iranian infertility Clinics in 2012-2014. The Iranian Journal of Obstetrics, Gynecology and Infertility.

[3] Kazem M, Ali A (2009). An overview of the epidemiology of primary infertility in Iran. J Reprod Infertil.

[4] Vafadar Z, Vanaki Z, Ebadi A (2017). An overview of the most prominent applied models of inter-professional education in health sciences in the world. Research in Medical Education.

[5] Karimi FZ, Taghipour A, Latifnejad Roudsari R, Kimiaee SA, Mazloum SR, Amirian M (2016). Psycho-social effects of male infertility in Iranian women: a qualitative study. The Iranian Journal of Obstetrics, Gynecology and Infertility.

[6] Rahmani Fard T, Kalantarkousheh SM, Faramarzi M (2017). The effect of mindfulness-based cognitive psychotherapy on quality of life in infertile women. Journal of Hayat.

[7] Khakpour M, Nejat H, Karimian F, Mehrafarid M, Mortazavi S, Chenari T (2017). Effect of fordyce happiness model on hardiness and marital adjustment in infertile couples. Journal of Nursing Education.

[8] Dizaji MB, Taghdisi MH, Solhi M, Hoseini SM, Shafieyan Z, Qorbani M (2014). Effects of educational intervention based on PRECEDE model on self care behaviors and control in patients with type 2 diabetes in 2012. J Diabetes Metab Disord.

[9] Abdoli S, Ashktorab T, Ahmadi F, Parvizy S, Dunning T (2011). Religion, faith and the empowerment process: stories of Iranian people with diabetes. Int J Nurs Pract.

[10] Nosek M, Kennedy HP, Gudmundsdottir M (2012). Distress during the menopause transition: a rich contextual analysis of midlife women’s narratives. Sage Open.

[11] Hasanpoor-Azghdy SB, Simbar M, Vedadhir A (2014). The emotional-psychological consequences of infertility among infertile women seeking treatment: results of a qualitative study. Iran J Reprod Med.

[12] Mardi A, Ebadi A, Shahbazi S, Esmaelzade Saeieh S, Behboodi Moghadam Z (2018). Factors influencing the use of contraceptives through the lens of teenage women: a qualitative study in Iran. BMC Public Health.

[13] Taherkhani S, Negarandeh R, Simbar M, Ahmadi F (2014). Iranian women’s experiences with intimate partner violence: a qualitative study. Health Promot Perspect.

[14] Graneheim UH, Lundman B (2004). Qualitative content analysis in nursing research: concepts, procedures and measures to achieve trustworthiness. Nurse Educ Today.

[15] Parvizi S, Mirbazegh F (2016). Ghasemzadeh Kakroudi F.A family-based model for Iranian women's health: a grounded theory study. J Public Health.

[16] Yazdkhasti M, Keshavarz M, Mahmoodi Z, Hosseini AF (2014). Self-directed learning and its impact on menopausal symptoms. Iran Red Crescent Med J.

[17] Thomson AA, Brown M, Zhang S, Stern E, Hahn PM, Reid RL (2016). Evaluating acquisition of knowledge about infertility using a whiteboard video. J Obstet Gynaecol Can.

[18] Kudesia R, Chernyak E, McAvey B (2017). Low fertility awareness in United States reproductive-aged women and medical trainees: creation and validation of the Fertility & Infertility Treatment Knowledge Score (FIT-KS). Fertil Steril.

[19] Swift BE, Liu KE (2014). The effect of age, ethnicity, and level of education on fertility awareness and duration of infertility. J Obstet Gynaecol Can.

[20] Hampton K, Mazza D (2015). Fertility-awareness knowledge, attitudes and practices of women attending general practice. Aust Fam Physician.

[21] Conceição C, Pedro J, Martins MV (2017). Effectiveness of a video intervention on fertility knowledge among university students: a randomised pre-test/post-test study. Eur J Contracept Reprod Health Care.

[22] Hampton KD, Newton JM, Parker R, Mazza D (2016). A qualitative study of the barriers and enablers to fertility-awareness education in general practice. J Adv Nurs.

[23] Dawdy K, Bonin K, Russell S, Ryzynski A, Harth T, Townsend C (2018). Developing and evaluating multimedia patient education tools to better prepare prostate-cancer patients for radiotherapy treatment (randomized Study). J Cancer Educ.

[24] Jurgenson JR, Jones EK, Haynes E, Green C, Thompson SC (2014). Exploring Australian Aboriginal Women’s experiences of menopause: a descriptive study. BMC Womens Health.

[25] Ahmadi B, Farzadi F, Dejman M, Vameghi M, Mohammadi F, Mohtashami B (2014). Farmehr model: Iranian women’s health conceptual framework. Hakim Health Sys Res.

[26] Mohamadirizi S, Fahami F, Bahadoran P (2014). Comparison of the effect of multimedia and illustrated booklet educational methods on women's knowledge of prenatal care. Iran J Nurs Midwifery Res.

[27] Sinclair P, Kable A, Levett-Jones T (2015). The effectiveness of internet-based e-learning on clinician behavior and patient outcomes: a systematic review protocol. JBI Database System Rev Implement Rep.

[28] Curtis K, Wiseman T, Kennedy B, Kourouche S, Goldsmith H (2016). Implementation and evaluation of a ward-based eLearning program for trauma patient management. J Trauma Nurs.

[29] Rimaz S, Merghati Khoii E, Zareie F, Shamsalizadeh N (2013). Iranian women's understandings of menopause and cultural scenarios. Journal of School of Public Health and Institute of Public Health Research.

[30] Yazdkhasti M, Keshavarz M, Khoei EM, Hosseini A, Esmaeilzadeh S, Pebdani MA (2012). The effect of support group method on quality of life in post-menopausal women. Iran J Public Health.

[31] van Empel IW, Nelen WL, Tepe ET, van Laarhoven EA, Verhaak CM, Kremer JA (2009). Weaknesses, strengths and needs in fertility care according to patients. Hum Reprod.

[32] Soleimani MA, Negarandeh R, Bastani F, Greysen R (2014). Disrupted social connectedness in people with Parkinson's disease. Br J Community Nurs.

[33] Mehrabi T, Alijanpoor Aghamaleki M, Hosseiny RS, Ziraki Dana A, Safaee Z (2014). A study on the relationship betweens spiritual well-being and quality of life in infertile women referred to infertillity centers in Isfahan. J Urmia Nurs Midwifery Fac.

